# Complicated Form of Medication Overuse Headache Is Severe Version of Chronic Migraine

**DOI:** 10.3390/jcm13133696

**Published:** 2024-06-25

**Authors:** Gülcan Göçmez Yılmaz, Reza Ghouri, Asena Ayça Özdemir, Aynur Özge

**Affiliations:** 1 Department of Neurology, Mersin City Training and Research Hospital, Mersin 33110, Turkey; gocmezgulcan@gmail.com; 2 Department of Neurology, Mersin University School of Medicine, Mersin 33110, Turkey; dr.rezaghouri@gmail.com; 3 Neuroscience ad Neurotechnology Center of Excellence (NÖROM), Gazi University, Ankara 06570, Turkey; 4 Department of Medical Education, Mersin University, Mersin 33343, Turkey; aycaozdemir@mersin.edu.tr

**Keywords:** chronic migraine, medication overuse headache, migraine complications, psychiatric comorbidities, headache disorders, migraine management, prophylactic therapy, pain localization, symptomatic treatment, headache recurrence

## Abstract

**Background**: MOH (medication overuse headache) is regarded as a complication of chronic migraines (CMs), with a general acknowledgment of reciprocal triggering between these two conditions. The present study aims to investigate the clinical parameters of relevance for the development of MOH among patients with CM, as well as for the subtype classification of MOHs. **Method**: We compared two groups of CM patients, with and without MOH, separated based on their demographic data and migraine characteristics. A subgroup of MOH accompanied by psychiatric co-morbidities (depression, anxiety, sleep disorder) was delineated, and the clinical features of relevance for the progression of MOH into the complicated state were evaluated. **Results**: The study revealed a higher prevalence of a family history of migraine in both the MOH and potentially complicated MOH subgroups (*p* < 0.001, *p* = 0.036), along with a higher prevalence of bilateral pain localization (*p* = 0.033, 0.021). Symptoms commonly associated with migraines, such as nausea, vomiting, photophobia, phonophobia, and osmophobia, were more common in both the MOH and potentially complicated MOH subgroups (*p* < 0.05). Furthermore, a positive correlation was found for the frequency (*p* < 0.001) and severity (*p* = 0.010) of migraine attacks and the duration of headaches (*p* = 0.007), atopy (*p* = 0.017), sleep disturbances (*p* = 0.011), and emotional stress (*p* = 0.022) in the MOH group. **Conclusion**: We found a positive correlation between the prevalence of MOH among patients with CM and a family history of migraines, higher frequency and intensity of headaches, bilateral manifestation, sleep disturbances, and emotional stress. Moreover, symptoms accompanying migraines were found to be more prevalent in individuals with MOH and potentially complicated MOH.

## 1. Introduction

Chronic migraines (CMs) are a significant cause of headache-related disability. The prevalence of chronic migraines is 1.4–2.2% in the general population and 8% among migraine patients [1,2]. CM patients, who have an important place among migraine patients, constitute a more treatment-resistant group, in which comorbid conditions are more common and are a significant cause of socioeconomic burden [2].

CMs are characterized by the persistence of migraine headaches for at least 3 months, 15 or more days per month, according to the International Classification of Headache Disorders (ICHD)-3 criteria [3]. CMs often evolve from poorly managed episodic migraine pain. Medication overuse headaches (MOHs) have been accepted as a complication accompanying CM headaches [4]. According to the ICHD-3 criteria, an MOH is defined as a secondary chronic headache that develops in patients who use symptomatic drugs for more than 3 months, more than 15 days a month for NSAIDs (non-steroidal anti-inflammatory drugs), and more than 10 days a month for opioid, ergotamine, and triptan group drugs [3,5].

MOH is divided into type 1 simple form and type 2 complex form. A type 1 MOH is defined by relatively short-term drug use, minimal psychiatric comorbidity, and no recurrence after drug withdrawal. Type 2 MOH is a more complex form with long-term drug use, the presence of multiple psychiatric comorbidities, and recurrences after discontinuation of drug overuse [6,7].

It is known that the most important risk factor for MOH is the presence of a previous primary headache, especially migraine [7]. The high frequency of headaches and the lack of effective prophylactic treatment trigger the use of symptomatic medication. Studies have shown that the intensive use of symptomatic treatment activates certain pain mechanisms that lead to the chronicization of pain [8,9]. In some studies, it has been noted that excessive use of symptomatic treatment, such as in osteoarticular diseases, does not lead to MOH, while in patients primarily with migraine, excessive medication usage is observed to be associated with headaches [10]. This also supports the development of an MOH as a complication of a migraine.

Our hypothesis aims to shed light on the divergence–convergence points of MOH development and its subtypes among CM patients, providing insights for clinicians regarding the clinical characteristics to consider during both short-term and long-term patient management.

## 2. Methods

The universe of the study included patients diagnosed with chronic migraines as per the criteria specified in the International Classification of Headache Disorders (ICHD-3), whose migraine data were recorded in the migraine headaches database upon their visit to the Neurology Department at Mersin University Faculty of Medicine between 2010 and 2022 [3]. As per the inclusion criteria, the universe included patients with the following characteristics: (i) 18–80 years of age; (ii) diagnosed with CM in line with the ICHD-3 diagnostic criteria for CM; and not diagnosed with (iii) dementia, (iv) psychosis, or (v) intellectual disability. On the other hand, the exclusion criteria included the following: (i) patients with headaches that do not meet the diagnostic criteria for migraines in the ICHD-3 beta and patients diagnosed with (ii) dementia, (iii) psychosis, or (iv) intellectual disability were excluded from the study sample.

Accordingly, 824 of the patients with CMs were found to be in the age range of 18 to 80 years. Among them, 143 were identified as meeting the diagnostic criteria for MOHs in the ICHD-3 [3].

This group of patients was first divided into two groups based on the presence or absence of MOH and then these two groups were compared in terms of age, gender, smoking (yes/no) and alcohol consumption (yes/no), a family history of headaches (yes/no), and their weekly duration of physical activity (at least 30 min once a week). This division was followed by queries to establish the monthly frequency, pain severity on the Visual Analog Scale (VAS), and number of years with migraine, with pain localization being characterized as unilateral, bilateral, and both unilateral and bilateral. The subsequent queries covered the presence or absence of symptoms accompanying the headaches, including nausea, vomiting, photophobia, phonophobia, osmophobia, allodinia, and autonomous symptoms, as well as the use of preventive medication. Finally, the groups with and without MOH were compared according to the presence or absence of comorbidities such as atopy, allergies, sleep disturbances, emotional stress, anxiety, depression, bruxism, and fibromyalgia.

As a sub-analysis in our study, subgroups were created as follows: patients with CMs and MOHs, 83 patients with an MOH and without psychiatric co-morbidities such as depression, anxiety, and sleep disorders, and 60 patients with a complicated MOH with psychiatric comorbidities. These subgroups were then compared based on demographic data and migraine characteristics. Furthermore, the study investigated the clinical parameters that might contribute to the progression of MOHs into the complicated form. The whole structure of the study was built in line with the STROBE Checklist.

The sub-analyses evaluated the demographics and clinical variables of relevance for the clinical phenotypes, with specific testing of the clinical parameters that potentially play a significant role in the distinctions of MOH sub-types.

### 2.1. Statistical Analysis

In our study, normality control of continuous variables was performed with the Shapiro–Wilk test. Comparisons between the two groups for variables that conformed to the normal distribution were evaluated with the Independent Sample *t*-test, and variables that did not conform to the normal distribution were evaluated with the Mann–Whitney test. The chi-squared and Fisher’s exact tests were used to analyze categorical variables. Multivariate Logistic Regression analysis was performed with variables found to be associated with MOH. Data analysis was performed in the TIBCO Statistica program.

### 2.2. Ethical Approval

The local ethics committee of Toros University approved the study with the Institutional Board Decision numbered 2023/98 on 27 September 2023.

## 3. Results

The present study included a total of 824 patients diagnosed with CMs, namely 143 with MOHs and 681 without MOHs. The findings indicated no significant difference in mean age between the groups with and without MOHs (*p* = 0.656). Among the patients with CMs, 138 (16.7%) were male and 686 (83.3%) were female. There was no significant difference in gender distribution between the groups with and without MOH (*p* = 0.468). An analysis of the groups in terms of smoking and alcohol consumption also found no significant difference between the groups (*p* = 0.663 and *p* = 0.146, respectively). A family history of migraine was found to be significantly more prevalent in the MOH group (*p* < 0.001). There was, however, no correlation between daily physical activity and MOHs (*p* = 0.014) (Table 1).

The part of the study concerning the clinical features of migraines showed a significantly higher frequency of attacks in individuals with MOHs (*p* < 0.01). Pain intensity was assessed using the Visual Analog Scale (VAS), revealing significantly elevated mean VAS scores in the MOH group (*p* = 0.010). Moreover, migraine headaches in the MOH group manifested more frequently with bilateral localization, and the migraine duration was observed to be prolonged in this group (*p* = 0.033, *p* = 0.007, respectively). Notably, accompanying symptoms such as nausea, vomiting, photophobia, phonophobia, osmophobia, allodynia, and autonomic symptoms were statistically more prevalent among patients with MOHs (*p* < 0.05). Additionally, a higher proportion of individuals with an MOH received prophylactic therapy compared to those without (*p* < 0.001, *p* = 0.03, respectively).

The investigation addressing comorbidities in patients with CM found that the MOH group exhibited a higher prevalence of atopy, sleep disturbances, and emotional stress (*p* = 0.017, 0.011, 0.022, respectively). However, no significant correlations were found for allergies, anxiety, depression, bruxism, or fibromyalgia as symptoms accompanying the migraines (Table 2).

**Figure 1:**
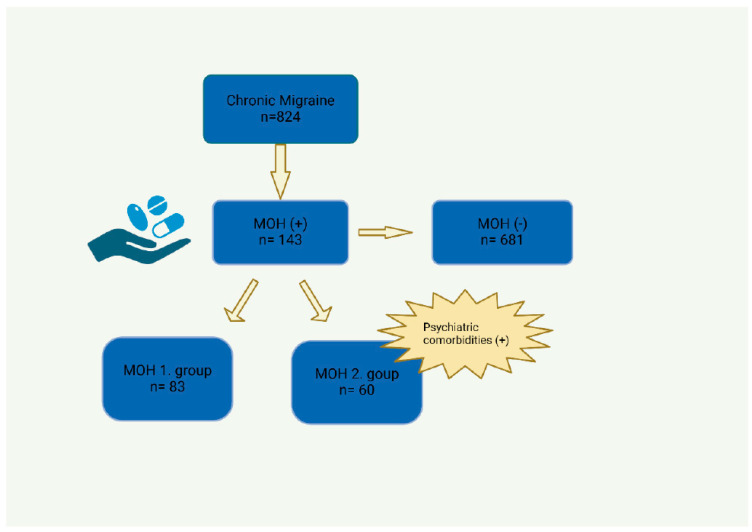
Flow chart of patient groups. (MOH: Medication overuse headache).

The study was conducted via a stepwise multivariate logistic regression analysis of all the variables regarding CMs and MOHs. Table 3 presents the variables under study, the analyses of which revealed the positive correlations of statistical significance. Accordingly, higher VAS scores were found to increase the likelihood of MOH (*p* = 0.046, (OR = 1.44; 95% CI = 1.01–2.05)). Pain frequency and bilateral pain localization were directly proportional to an increase in the development of MOHs (*p* = 0.010, (OR = 1.07; 95% CI = 1.02–1.13), *p* = 0.013, (OR = 5.92; 95% CI = 1.46–24.04), respectively). Positive correlations were also observed between MOHs and phonophobia, photophobia, and allodynia (*p* = 0.002, (OR = 55.70; 95% CI = 4.65–667.10), *p* = 0.033, (OR = 0.21; 95% CI = 0.05–0.89), respectively). A statistically significant positive result was also detected for allodynia (*p* = 0.041, (OR = 2.68; 95% CI = 1.04–6.91)). Finally, a statistically significant correlation was found for fibromyalgia among the comorbidities (*p* = 0.035, (OR = 0.32; 95% CI = 0.11–0.92)). We have created a flow chart of patient groups in Figure 1 that summarizes the distinction of patient groups.

Table 4 separates the patients with MOH into two groups, namely MOH-1, comprising individuals without psychiatric comorbidities (anxiety, depression, or sleep disorders), and MOH-2, consisting of those with any of the aforementioned psychiatric comorbidities, for comparisons based on the clinical features of migraines. No statistically significant differences were observed in age, gender, smoking status, alcohol consumption, or physical activity levels between the two groups (*p* = 0.260, 0.813, 0.928, 0.00, 0.770, respectively). However, a higher prevalence of a family history of migraines was noted in the group of patients with potentially complicated MOHs, along with a higher prevalence of bilateral pain localization (*p* = 0.036, 0.021, respectively). No significant differences were found between the MOH groups regarding attack frequency, pain severity (VAS), and migraine duration (*p* = 0.129, 0.356, 0.255, respectively). No statistically significant relationship was found in the two groups with allodynia and preventive therapy (0063, 0.864, respectively). However, symptoms that might accompany migraines such as nausea, vomiting, photophobia, phonophobia, dizziness, osmophobia, and autonomic symptoms were more common in the group of patients with potentially complicated MOHs, with findings of statistical significance (*p* < 0.005).

## 4. Discussion

Medication overuse is recognized as a factor that contributes to the progression of migraines to the chronic form [11,12,13]. An MOH is regarded as a complication of CMs, with reciprocal triggering between these two conditions being acknowledged in the literature [14]. The progression to an MOH complicates the management of cases with CMs and often leads to post-treatment recurrence, making it a crucial goal for the field to identify the factors associated with the condition. The present study encompassed an investigation of the various clinical features and comorbidities in CM patients to provide insights into the development of MOHs. Furthermore, the authors explored the patient characteristics and clinical features of migraines that may play a role in the progression of a case to the more complicated form known as type-2 MOH.

The present study, involving 824 patients with CMs, found no significant differences between the groups with and without MOHs in terms of age, gender, alcohol consumption, and smoking. However, an examination of the characteristics of migraines revealed that CM patients with MOHs exhibited higher attack frequencies and scored higher on the VAS. This finding of the study is in contrast with the findings of a longitudinal population-based study conducted in Italy, where a higher prevalence of MOHs and chronic headaches was observed among women, with no positive correlations established in terms of migraine characteristics [15]. However, another study underlined the frequency of headaches as an important risk factor for the development of CM-MOHs [16].

The present study also revealed a higher prevalence of bilateral pain localization and a prolonged migraine duration for the MOH group. Additionally, a family history of migraines was more common in patients with MOHs. In addition to this conclusion, in some studies, it has been suggested that the presence of a family history of MOHs also increases the development of an MOH [7]. Furthermore, in our study, it was found that preventive therapies were used more frequently in MOHs. Similar to the findings of the present study, another study examining patients with CM established a higher prevalence of preventive therapies for migraines among patients with MOHs [17]. It is of note that this study also established a significantly younger age of migraine onset in patients with an MOH [17].

On the other hand, a comparative study involving patients with episodic migraines and CMs found no significant correlation between a family history of migraines and CM-MOH. However, CM-MOH patients exhibited an earlier onset age of migraines and a higher prevalence of receiving at least one preventive therapy. Additionally, CM patients with MOH showed a higher prevalence of depression, a history of head trauma, insomnia, and childhood trauma among the comorbidities examined in this study [16].

Several psychosocial factors are known to be associated with the development of MOH [5]. In the present study, a positive correlation has been established between MOHs and comorbidities such as atopy, sleep disturbances, and emotional stress. However, no significant correlations were found for a history of allergies, anxiety, depression, bruxism, or fibromyalgia. A similar study focusing on psychiatric comorbidities revealed that lifetime anxiety and mood disorders were more prevalent in MOH patients compared to the control group [18]. Furthermore, this study also examined personality disorders and a family history of substance use, which were found to increase the risk of MOH development [18,19]. Another study investigating the progression of migraines to MOHs reported a higher prevalence of anxiety, depression, mood disorders, and psychoactive substance use among individuals with an MOH [20,21]. In a study that evaluated the development of MOHs and included CM patients, which was designed similarly to our study, no significant correlation was found between MOHs and anxiety, depression, and poor sleep [22].

Moreover, a significant correlation was reported between CMs with MOHs and allodynia. Allodynia, a finding often accompanying CMs, was suggested to represent a clinical marker for the progression to a chronic state [22,23]. It has been suggested that cortical spreading depression and increased CGRP, which are mechanisms in migraine pathogenesis, may play a role in the pathogenesis of MOHs, but central sensitization is thought to be the major factor [24]. Allodynia, one of the accompanying symptoms of migraines, is also accepted as a symptom that develops as a result of central sensitization. A study focusing on patients with CMs accompanied by MOHs revealed that the absence of allodynia served as a positive indicator following the discontinuation of medication overuse [23].

Recent findings have shown that some drugs can have transient effects of peripheral vasoconstriction or lowering systemic pressure levels. The abuse of these drugs may lead to paradoxical effects at the level of the extra-cranial vessels, which have been described as trigger points for migraine attacks in some patients. This effect is particularly relevant given the abnormalities found in these vessels in recent studies. Highlighting these mechanisms can provide a more comprehensive understanding of the factors contributing to the development of medication overuse headache (MOH) and its complications.

In particular, a study referenced by Raposio E et al. detailed how the misuse of certain medications can lead to significant changes in the extra-cranial vasculature, thereby acting as a potential trigger for migraine attacks [25]. Understanding these vascular changes is crucial for developing more effective treatment strategies and preventing the progression of chronic migraine (CM) to MOH.

The present study also investigated a subgroup of patients with potentially complicated MOHs and found a higher prevalence of a family history of migraines and more frequent bilateral localization of migraine pain in this patient group. Moreover, both patients with MOH and those in the potentially complicated MOH group exhibited a higher frequency of symptoms accompanying the migraines, such as nausea, vomiting, photophobia, phonophobia, and autonomic symptoms, compared to those without MOHs.

CMs present a subset within the population of patients with migraines that is characterized by a greater resistance to treatment. The definition of MOHs as a sub-type of CMs further complicates headache management. However, the reportedly high frequency of recurrence underlines the inadequacy of discontinuing medication overuse as a response to the onset of MOH. Progression of MOH to the more complicated form depends on the presence of psychiatric comorbidities and recurrence [26]. Therefore, the present study pursued a secondary aim of attempting to identify the clinical features of migraines associated with the potentially complicated form of the condition. The most effective response to MOHs in cases with CMs commonly relies on a preventive approach based on the identification of the clinical signs before the onset of medication overuse and progression to the more complicated form.

One notable aspect of the present study stems from its extensive longitudinal observation of a substantially larger group of patients with CMs compared to similar studies in the literature [16]. Furthermore, the present investigation comprehensively addresses the clinical data for both migraines and the comorbidities associated with the onset of MOHs.

The study’s findings should be interpreted with care in light of several limitations. Firstly, one limitation of the present research is represented by the lack of follow-up data concerning recurrences following the discontinuation of medication overuse among patients with MOHs. Longitudinal studies spanning extended periods, integrating detoxification processes and recurrence evaluations, are warranted to better understand the trajectory of MOH development in CM patients. Additionally, while the study assessed depression, anxiety, and sleep disorders, other potential psychiatric factors influencing medication overuse may not have been fully explored. Moreover, the study’s single-center design raises questions about the generalizability of findings to broader populations with chronic migraine and medication overuse headaches. Furthermore, the underrepresentation of type-2 medication overuse headache cases limits the understanding of this subtype’s clinical characteristics and risk factors. Lastly, reliance on self-reported data introduces the possibility of recall bias, highlighting the need for future studies to incorporate objective measures for enhanced accuracy. These limitations underscore the importance of further research to address gaps in knowledge and improve interventions for medication overuse headaches among chronic migraine patients.

The relationship between MOH and chronic migraine is complex, and while our study indicates that CM patients with MOHs exhibit a higher proportion of migraine characteristics and psychiatric disorders, it is important to recognize that these conditions may also be present in other forms of headaches or due to comorbid mental illnesses. The classification of a complicated MOH as a severe version of CMs remains a topic that warrants further investigation. Migraine patients, particularly those with CM, are known to be prone to comorbidities such as anxiety and depression, which complicates the clinical picture.

Our findings suggest significant clinical implications, yet it is crucial to consider the broader context of comorbid conditions. Future studies should aim to delineate the specific contributions of these comorbidities to the severity and classification of MOHs in CM patients. Understanding the interplay between these factors will help in developing more targeted and effective management strategies.

The findings of this study have several implications for clinical practice and future research. Firstly, understanding the long-term patterns of medication overuse post discontinuation is crucial for tailoring effective management strategies and reducing recurrence rates among chronic migraine patients. Clinicians should consider incorporating regular follow-up assessments to monitor medication use and evaluate the effectiveness of interventions over time. Additionally, the identification of psychiatric comorbidities associated with medication overuse highlights the importance of comprehensive psychiatric evaluation and integrated treatment approaches in managing chronic migraine. Moreover, while the study provides valuable insights into medication overuse headaches, further research is warranted to explore the generalizability of findings across diverse patient populations and healthcare settings. Future studies could also delve into the underlying mechanisms driving medication overuse and assess the efficacy of targeted interventions in mitigating its impact on migraine severity and disability. By addressing these implications, healthcare providers can optimize patient care and contribute to the development of more effective strategies for managing medication overuse headaches in chronic migraines.

## 5. Conclusions

The present study established positive correlations between MOHs and a family history of migraines, pain frequency and severity, bilateral pain localization, sleep disturbances, and emotional stress. In addition, patients with MOHs and potentially complicated MOHs exhibited a higher prevalence of accompanying migraine symptoms such as nausea, vomiting, photophobia, phonophobia, osmophobia, autonomic symptoms. The present study thus sheds light on the progression of CMs to MOHs, and the follow-up of patients with migraines with consideration for the characteristics of patients predisposed to the more complicated form of MOH and the evaluation of clinical data.

## 6. Key Messages

1. A medication overuse headache (MOH) is a frequent complication of chronic migraine (CMs) influenced by similar triggers and symptoms.
2. Complicated MOHs are associated with significant psychiatric comorbidities, highlighting the need for holistic treatment approaches.
3. A family history of migraines increases the likelihood of developing an MOH.
4. Characteristics such as high frequency, severe pain, and bilateral localization are closely linked to MOH development.
5. MOH patients often experience more intense migraine symptoms, such as nausea and photophobia.
6. Prophylactic treatments are more commonly used among MOH patients, indicating challenging management needs.
7. Early detection of MOH can lead to better management and potentially prevent its progression.
8. Comorbid conditions like sleep disturbances and emotional stress are prevalent in MOH patients and must be addressed in treatment.
9. Managing an MOH requires a multidisciplinary approach due to its complexity and the variety of symptoms and comorbidities involved.
10. The study’s findings stress the importance of further research to better understand MOHs and improve treatment strategies.

## Figures and Tables

**Table 1:** Comparison of epidemiological data and migraine characteristics in CM patients with and without an MOH.

	CM with MOH	CM without MOH	*p*
Age, years (mean, SD)	41.46 (11.81)	41.98 (15.5)	0.656
-Female -Male	122 (85.3) 21 (14.7)	564 (82.8) 117 (17.2)	0.468
Smoking, n (%)	21 (14.7)	110 (16.2)	0.663
Alcohol consumption, n (%)	0 (0)	14 (2.1)	0.146
Family history of migraine, n (%)	64 (44.8)	202 (29.7)	**<0.001**
Physical activity, n (%)	22 (15.4)	59 (8.7)	0.014
Frequency of attacks, day/months, mean (SD)	20.18 (8.66)	15.56 (9.62)	**<0.001**
VAS, (mean, SD)	8.52 (1.46)	7.87 (2.09)	**0.010**
Pain Localization, n (%) -Unilateral -Bilateral -Unilateral + Bilateral	40 (60) 10 (10.2) 16 (24.2)	156 (76. 8) 16 (7.9) 31 (15.3)	**0.033**
Migraine duration, month Mean (SD)	194. 48 (134.26)	159. 59 (137.16)	**0.007**
Pain acompanying symptoms			
Nausea, n (%)	84 (58.7)	309 (45.4)	**0.004**
Vomiting, n (%)	54 (37.8)	182 (26.7)	**0.008**
Photophobia, n (%)	81 (56.6)	295 (43.3)	**0.004**
Phonophobia, n (%)	87 (60.8)	305 (44.8)	**<0.001**
Osmophobia, n (%)	75 (52.4)	246 (36.1)	**<0.001**
Allodynia, n (%)	32 (22.4)	102 (15)	**0.029**
Autonomic Symtoms, n (%)	13 (9.1)	29 (4.3)	**0.017**
Prophylaxis, n (%)	113 (79.0)	453 (66.5)	**0.003**

CM: Chronic migraine, MOH: Medication overuse headache, VAS: Visual Analog Scale, SD: Standard deviation.

**Table 2:** Comparison of co-morbid conditions in CM patients with and without MOHs.

	MOH+	MOH−	*p*
Atopy, n (%)	34 (23.8)	106 (15.6)	**0.017**
Allergies, n (%)	6 (4.2)	37 (5.4)	0.545
Sleep disturbances, n (%)	50 (35.0)	168 (24.7)	**0.011**
Emotional stress, n (%)	40 (28)	132 (19.4)	**0.022**
Anxiety, n (%)	18 (12.6)	117 (17.2)	0.177
Depression, n (%)	10 (7.0)	44 (6.5)	0.815
Bruxizm, n (%)	9 (6.3)	45 (6.6)	0.890
Fibromyalgia, n (%)	29 (20.3)	130 (19.1)	0.743

**Table 3:** MOH-related factors in CM patients according to multivariate logistic regression analysis results.

	OR	95% C.I. for OR		*p*
		Lower	Upper	
VAS	1.44	1.01	2.05	**0.046**
Frequency of attacks	1.07	1.02	1.13	**0.010**
Pain Localization (bilateral)	5.92	1.46	24.04	**0.013**
Phonophobia	55.70	4.65	667.10	**0.002**
Photophobia	0.21	0.05	0.89	**0.033**
Allodynia	2.68	1.04	6.91	**0.041**
Fibromyalgia	0.32	0.11	0.92	**0.035**

MOH: Medication overuse headache, CM: Chronic migraine, OR: Odds Ratio, VAS: Visual Analog Scale.

**Table 4:** Comparison of characteristics between MOH group-1 without psychiatric co-morbidities and possibly complicated MOH group-2.

	MOH Grup-1	MOH Grup-2	*p*
Age, years (mean, SD)	40.49 (10.3)	42. 77 (13.57)	0.260
Female -Male	70 (84.3) 13 (15.7)	52 (86.7) 8 (13.3)	0.813
Smoking, n (%)	12 (14.5)	9 (15.0)	0.928
Alcohol consumption, n (%)	83 (100)	60 (100)	0.00
Family history of migraine, n (%)	31 (37.3)	33 (55.0)	**0.036**
Physical activity, n (%)	9 (10.8)	13 (21.7)	0.770
Frequency of attacks, day/months, mean (SD)	18.56 (8.66)	21.3 (8.56)	0.129
VAS, mean (SD)	8.52 (1.88)	8.52 (1.09)	0.356
Pain Localization, n (%) -Unilateral -Bilateral -Unilateral + Bilateral	22 (78.6) 1 (3.6) 5 (17.9)	18 (47.4) 9 (23.7) 11 (28.9)	**0.021**
Migraine duration, month Mean, SD	210.98 (136.4)	181.81 (132.53)	0.255
Pain acompanying symptoms			
Nausea, n (%)	36 (43.4)	48 (80.0)	**<0.001**
Vomiting, n (%)	25 (30.1)	29 (48.3)	**0.027**
Photophobia, n (%)	35 (42.2)	46 (76.7)	**<0.001**
Phonophobia, n (%)	41 (49.4)	46 (76.7)	**0.001**
Osmophobia, n (%)	34 (41.0)	41 (68.3)	**0.001**
Allodynia, n (%)	14 (16.9)	18 (30.0)	0.063
Autonomic Symtoms, n (%)	4 (4.8)	9 (15.0)	**0.044**
Prophylaxis, n (%)	66 (79.5)	47 (78.3)	0.864

MOH: Medication overuse headache, SD: Standard deviation, VAS: Visual Analog Scale.

## Data Availability

Data is contained within the article.
